# Two new genera and three new species of Epipaschiinae Meyrick from China (Lepidoptera, Pyralidae)

**DOI:** 10.3897/zookeys.722.12362

**Published:** 2017-12-13

**Authors:** Mingqiang Wang, Fuqiang Chen, Chaodong Zhu, Chunsheng Wu

**Affiliations:** 1 Key Laboratory of Zoological Systematics and Evolution, Institute of Zoology, Chinese Academy of Sciences, Beijing 100101, P.R. China; 2 University of Chinese Academy of Sciences, Beijing, 100049, P. R. China

**Keywords:** New genus, new species, Pyraloidea, snout moth, taxonomy

## Abstract

Two new genera of Epipaschiinae are described. The genus *Arcanusa* Wang, Chen & Wu, **gen. n.** is established for *Ar. apexiarcanusa* Wang, Chen & Wu, **sp. n.** and *Ar. sinuosa* (Moore, 1888), **comb. n.**, described in *Scopocera* Moore, 1888 (junior synonym of *Stericta* Lederer, 1863). The female genitalia of *Ar. sinuosa* (Moore, 1888), **comb. n.** are described for the first time. *Androconia* Wang, Chen & Wu, **gen. n.** is erected, including two new species, *An. rallusa* Wang, Chen & Wu, **sp. n.** and *An. morulusa* Wang, Chen & Wu, **sp. n.** Illustrations of all adults and their genitalia, and a key to the two new genera are provided.

## Introduction

The subfamily Epipaschiinae Meyrick, 1884, includes more than 700 species distributed all over the world (Solis 1992), and embodies 93 genera currently (Nuss et al. 2003–2017). The post taxonomic works of were mainly published from the late 19^th^ century to the early 20^th^ century, while little work was done in the middle to the later 20^th^ century. In recent decades, ten new genera and some new species were reported from the Neotropical, Paleotropical, and Afrotropical regions (Solis 1991, 1993; Shaffer and Solis 1994; Mey 2011). Few works were published from the Palaearctic and Oriental regions, except some new species described by Inoue and Sasaki (1995), Liu et al. (2016), and Wang et al. (2017a, b).

During our work, some species were identified not attributed to any known epipaschiine genus. Thus, two new genera, *Arcanusa* Wang, Chen & Wu, gen. n. and *Androconia* Wang, Chen & Wu, gen. n., are proposed for them here, including two species each.

## Materials and methods

The specimens examined and the types of the new species are deposited in the collection of the Institute of Zoology, Chinese Academy of Sciences (IZCAS), Beijing, P. R. China. The specimens were collected by light trap. The photographs of moths and their genitalia were taken with a NIKON D7000 digital camera connected to a NIKON SMZ 1500 stereomicroscope. The images were adjusted by Adobe Photoshop CS5 and Adobe Illustrator CS6 software, and the distribution maps were made by ArcGIS 10.2 software. Methods of dissection, morphometrics, and terminology follow Wang et al. (2003) and Slamka (2006, 2008).

## Taxonomy

### Arcanusa

Taxon classificationAnimaliaLepidopteraPyralidae

Wang, Chen & Wu gen. n.

http://zoobank.org/0BA9E0CF-030E-4272-B80E-B281568012F9

#### Type species.

*Arcanusa apexiarcanusa* Wang, Chen & Wu, sp. n.

#### Diagnosis.

*Arcanusa* gen. n. differs from all known Epipaschiinae by the juxta of the male genitalia, which lateral lobes joined distally. The new genus is very similar to *Lista* Walker, 1859 based on the long hair-like scales at the base of the forewing and the similar pattern on both pairs of wings. In the male genitalia, the base of the sacculus is usually expanded and extended into a sclerotized spine or process, which is similar to that found in the genus *Lista*. However, the new genus can be distinguished by its dark scales on both wings while the species of *Lista* are covered with brighter scales. The new genus is also similar to *Coenodomus* Walsingham, 1888 in wing pattern and sclerotized process of the sacculus, but it can be distinguished easily by its filiform antennae in the male. In addition, the two genera have minute differences on the stalked R_3-5_ of the forewing.

#### Description.

Medium sized to Pyralidae (14.5–15.5 mm in forewing length). Head covered with dense scales; labial palpus upturned, third segment slender and obviously pointed; antenna filiform, male with a scape extension covered with dense scales. Forewing with distinct antemedial and postmedial lines. Hindwing with similar pattern as in forewing, but paler than forewing.

**Venation** (Fig. 22). In forewing, Sc reaching 1/2 of costa; R_1_ arising from 1/2 of upper margin of cell; R_2_ arising before upper angle of cell; R_3-5_ and M_1_ from upper angle of cell; R_3+4_ stalked with R_5_ at mid-length; M_2_ and M_3_ from lower angle of cell, CuA_1_ and CuA_2_ nearly parallel; 1A+2A anastomosed at base. In hindwing, Sc+R_1_ and Rs adjacent in middle of Sc+R_1_; Rs shortly stalked with M_1_; M_2_ and M_3_ separated from lower angle of cell; CuA_1_ and CuA_2_ nearly parallel; three A veins present.

**Male genitalia.** Uncus expanded. Gnathos pointed apically. Valval costa sclerotized; sacculus expanded at base, with a spine or process in middle. Juxta with two strongly sclerotized lateral lobes joined in apex. Phallus slender, slightly curved, spine-like cornuti present.

**Female genitalia.** Ovipositor expanded obviously. Apophysis anterioris lightly longer than apophysis posterioris. Antrum lightly sclerotized. Ductus bursae membranous. Corpus bursae rounded, signa consisting of two sclerotized incurved plates.

#### Distribution.

China, India (Fig. 24).

#### Etymology.

The generic name is derived from Latin “*Arcanus*” (= closed), in reference to the lateral lobes of juxta joined distally in male genitalia.

#### Key to species of *Arcanusa* Wang, Chen & Wu, gen. n.

**Table:** 

1	Hindwing with black scales on central area, sacculus with a pointed process in middle (Figs 2, 8)	***Arcanusa sinuosa* comb. n.**
–	Hindwing with pale yellow scales on central area, sacculus with a slightly bifurcated process in middle (Figs 1, 7)	***Arcanusa apexiarcanusa* Wang, Chen & Wu, sp. n.**

### Arcanusa apexiarcanusa

Taxon classificationAnimaliaLepidopteraPyralidae

Wang, Chen & Wu sp. n.

http://zoobank.org/77E22945-54B3-4B8F-9E35-267B05BB2D86

Figs 1 , 7 , 14

#### Differential diagnosis.

The new species is very similar to *Ar. sinuosa* comb. n. in external characters, especially the forewing. However, the hindwing is obviously paler than the forewing in the new species, while lightly paler in *Ar. sinuosa*. In male genitalia, *Ar. apexiarcanusa* Wang, Chen & Wu, sp. n. has the gnathos beaked and the sacculus with a bifurcated stick-like process while *Ar. sinuosa* has the gnathos trifurcate with a spine-like apex and the sacculus has a spine-like process. In addition, there are more minute spines on the juxta of the new species than on that of *Ar. sinuosa*.

**Figures 1–6.**
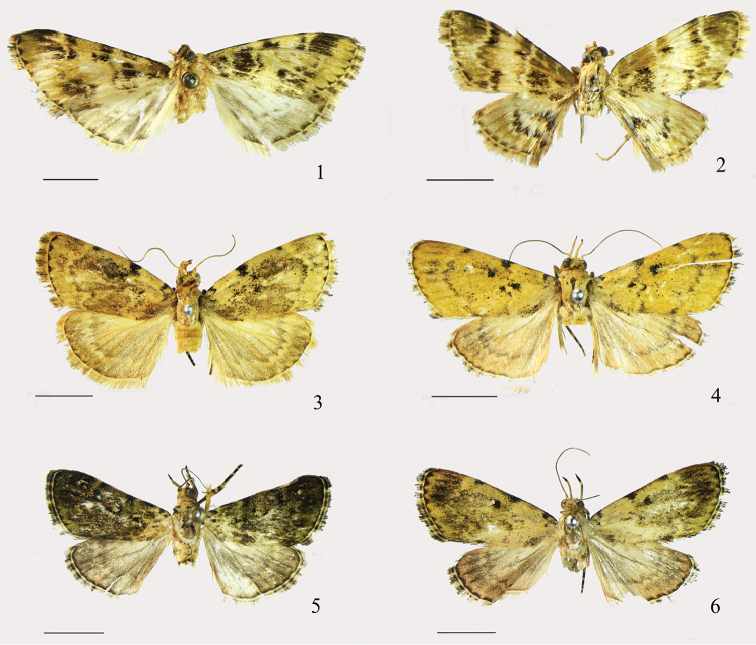
Adults. **1** *Arcanusa apexiarcanusa* Wang, Chen & Wu, sp. n., male, holotype **2** *Ar. sinuosa* (Moore, 1888), comb. n., male **3** *Androconia rallusa* Wang, Chen & Wu, sp. n., male, paratype **4** *An. rallusa* Wang, Chen & Wu, sp. n., female, paratype **5** *An. morulusa* Wang, Chen & Wu, sp. n., male, holotype **6** *An. morulusa* Wang, Chen & Wu, sp. n., female, paratype. Scale bars: 5.0 mm.

#### Description.

Adult male. Forewing length 15.5 mm (n=1). Head yellow mixed with fuscous; first and second segments of labial palpus with fuscous scales on outer side and pale yellow scales on inner side; third segment with pale brown scales; antenna yellow, scape extension fuscous, mixed with black scales on outer side and pale yellow scales on inner side; apical 2/3 of scape extension with yellow hair-like scales. Thorax mixed with yellow and small number of brown scales. Forewing covered with pale yellow, yellow and black scales; base black mixed with yellow scales, antemedial line black, waved, a black patch above discocellular; central area suffused with yellow scales; postmedial line black, waved, arched outward medially; outer area covered with yellow scales; cilia mixed with yellow and brown. Hindwing pale fuscous, with a blurry waved postmedial line.

**Male genitalia.** Uncus broad, slightly expanded at apex, densely suffused with setae laterally, apex truncated. Gnathos strongly sclerotized, beaked. Valva widening from base to apex, apex expanded; sacculus with stick-like sclerotized process in middle, bifurcated at apex. Juxta with two strongly sclerotized lateral lobes joined in apex, apex with minute spines. Phallus cylindrical, curved slightly, with many spinules laterally.

**Female genitalia.** Unknown.

#### Holotype.

♂, **Yunnan**: Jinghong, 2700 m, 4.VII.1980, gen. slide. no. Ep537 (IZCAS).

#### Distribution.

Yunnan.

#### Etymology.

The specific name is derived from the Latin “*apex*” (= apex) and “*arcanus*” (= closed) in accordance with the lateral lobes of juxta joined distally in the male genitalia.

### Arcanusa sinuosa

Taxon classificationAnimaliaLepidopteraPyralidae

(Moore, 1888) comb. n.

Figs 2 , 8 , 11 , 15

Scopocera sinuosa Moore, 1888: see Moore 1888: 203. Type Locality: India (Darjeeling).
Stericta sinuosa (Moore): see Hampson 1896: 122; Wang et al. 2003: 117; Rong and Li 2017: 470.

#### Differential diagnosis.

The species is unique in both the external and genital characters than other species of *Stericta* by its distinct antemedial line and the different juxta. The species is similar to *Ar. apexiarcanusa* Wang, Chen & Wu, sp. n. Their differences are described above.

#### Redescription.

Adult. Forewing length 11.0–15.0 mm (n=7). Head yellow mixed with fuscous; labial palpus first segment of labial palpus with pale brown scales, second segment with more fuscous scales than first; third segment with pale yellow scales, apically exceeding metathorax; antenna suffused with yellowish-brown scales. One-third length of scape extension at base with yellow and mixed with black scales; 2/3 length of scape extension closer apex with yellow and black hair-like scales. Thorax mixed with yellow and small number of blackish-brown scales. Forewing covered with yellow, yellowish-brown and black scales; base covered with black scales, antemedial line black, waved, a black patch located on discocellular; central area suffused with pale yellow scales; postmedial line black, waved, arched outward medially; outer area covered with yellowish-brown scales; cilia mixed yellow and brown. Hindwing paler than forewing, with distinct waved postmedial line and more or less black basal area.

**Male genitalia.** Uncus broad, expanded at apex, densely suffused with setae laterally. Gnathos strongly sclerotized, apex with trifurcate spine-like process. Valva nearly the same width from base to apex, apex obliquely truncated; costa strongly sclerotized, apex swollen; sacculus expanded at base, with a spine-like process in middle. Juxta with two strongly sclerotized lobes joined at apex, apex with minute spines. Phallus cylindrical, with many slender spines distally, curved slightly.

**Female genitalia.** Ovipositor obviously expanded. Apophysis anterioris lightly longer than apophysis posterioris. Antrum lightly sclerotized. Ductus bursae short, membranous. Corpus bursae rounded, signa consisting of two sclerotized incurved plates.

#### Material examined.

**Fujian**: Wuyishan, Sangang, 1♂, gen. slide. no. Ep704 (IZCAS); Chong’anxingcun, Sangang, 740 m, 1♀, 17.VI.1960, Zhang Yiran, gen. slide. no. Ep538 (IZCAS). **Hainan**: Jianfengling, 1♂, 23.X.1981, Gu Maobin (IZCAS). **Yunnan**: Jinping, Hetouzai, 1600 m, 1♂, 13.V.1956, Huang Keren, gen. slide. no. Ep112 (IZCAS); Pingbian, Daweishan, 1500 m, 1♂, 20.VI.1956, Huang Keren, gen. slide. no. Ep705 (IZCAS); Pingbian, 1200–1600 m, 1♀, 18.VII.1958, Huang Keren (IZCAS); Xishuangbanna, Menghai, 1500 m, 1♂, 20.VI.1956, Wang Shuyong (IZCAS).

**Figures 7–10.**
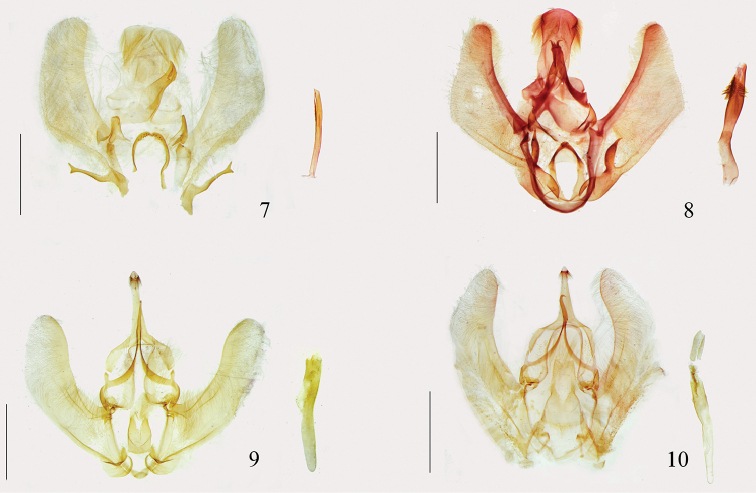
Male genitalia. **7** *Arcanusa apexiarcanusa* Wang, Chen & Wu, sp. n., holotype, gen. slide no. Ep537 **8** *Ar. sinuosa* (Moore, 1888), comb. n., gen. slide no. Ep112 **9** *Androconia rallusa* Wang, Chen & Wu, sp. n., holotype, gen. slide no. Ep680 **10** *An. morulusa* Wang, Chen & Wu, sp. n., holotype, gen. slide no. Ep691. Scale bars: 1.0 mm.

#### Distribution.

China (Fujian, Hainan, Yunnan), Bhutan, India.

#### Remarks.

This species was firstly reported in China (Wuyishan Nature Reserve, Fujian) by Wang et al. (2003). We reexamined their voucher material from Fujian (Ep704), which are in accordance with the original description of *Stericta sinuosa* (Moore, 1888). The species was originally described in *Scopocera* (junior synonym of *Stericta*). However, the species is closer to *Lista* than *Stericta*, because it does not have hairs on the outer side of mid-leg tibiae and has quite different genitalia to those of *Lista*. Thus, a new genus is erected to place the species and a new species, *Ar. apexiarcanusa* Wang, Chen & Wu, sp. n. In addition, the female is reported for the first time here.

### Androconia

Taxon classificationAnimaliaLepidopteraPyralidae

Wang, Chen & Wu gen. n.

http://zoobank.org/5B123A0D-CDD7-4F54-BB5C-D65A41CB5A7A

#### Type species.

*Androconia rallusa* Wang, Chen & Wu, sp. n.

#### Diagnosis.

The new genus is similar to *Stericta* Lederer, 1863 based on the wing pattern, especially the black discocellular spot on the forewing. However, it can be easily distinguished from *Stericta* by its males having an androconium at the discocellular cell of the forewing and the slender uncus which is obviously thinner than that of the other genera of Epipaschiinae.

#### Description.

Medium sized to Pyralidae (9.0–12.0 mm in forewing length). Head covered with dense scales; labial palpus upturned, third segment slender and pointed obviously; antenna filiform. Forewing with an androconium at discocellular cell in male, two black spots located at basal and terminal cell, respectively; antemedial and postmedial lines wavy, distinct, hindwings suffused with pale red scales.

**Venation** (Fig. 23). In forewing, Sc reaching 2/3 of costa; R_1_ arising from 2/3 of upper angle of cell; R_2_ arising before upper angle of cell; R_3-5_ and M_1_ from upper angle of cell and short stalked at base; R_3+4_ stalked with R_5_ at mid-length; M_2_ and M_3_ from lower angle of cell in same point; CuA_1_ and CuA_2_ nearly parallel; 1A+2A anastomosed at base. In hindwing, Sc+R_1_ and Rs connected at middle of Sc+R_1_; Rs shortly stalked with M_1_; M_2_ and M_3_ separated from lower angle of cell in same point; CuA_1_ and CuA_2_ nearly parallel; three A veins present.

**Male genitalia.** Uncus slender, densely setose. Gnathos with slender lateral arms, apex hooked. Valva slender, costa lightly sclerotized; sacculus swollen and warped in base. Juxta bifurcated. Phallus slender.

**Female genitalia.** Ovipositor covered with dense setae. Apophysis anterioris nearly as same length as apophysis posterioris. Antrum and ductus bursae membranous. Corpus bursae nearly elliptic, with two signa.

#### Distribution.

China (Fig. 24).

#### Etymology.

The generic name is in accordance with the androconium of the male forewing, derived from the Greek “*andro*” and “*konos*”.

#### Remarks.

The species of the new genus is sexually dimorphic in color. Although both sexes have the same wing pattern on both wings, the female has the forewing with less black scales and androconium absent, and the hindwing looks redder than the forewing.

#### Key to species of *Androconia* Wang, Chen & Wu, gen. n.

**Table:** 

1	Forewing suffused with yellow scales. Male with juxta nearly same width from base to apex and cornuti absent (Figs 3, 9); female genitalia with ductus bursae and ductus seminalis relatively narrow	***Androconia rallusa* Wang, Chen & Wu, sp. n.**
–	Forewing suffused with fuscous scales. Male genitalia with juxta constricted from base to apex and an irregular cornutus (Figs 5, 10); female genitalia with ductus bursae and ductus seminalis relatively broad	***Androconia morulusa* Wang, Chen & Wu, sp. n.**

### Androconia rallusa

Taxon classificationAnimaliaLepidopteraPyralidae

Wang, Chen & Wu sp. n.

http://zoobank.org/7BFC4AE6-CFC3-47BC-82D3-CBB97375A692

Figs 3–4 , 9 , 12 , 16–17 , 20–21

#### Differential diagnosis.

The species is similar to *An. morulusa* Wang, Chen & Wu, sp. n. in external characters. However, *An. morulusa* Wang, Chen & Wu, sp. n. has darker wings than *An. rallusa* Wang, Chen & Wu, sp. n. In the male genitalia, the juxta of *An. rallusa* Wang, Chen & Wu, sp. n. is thinner than *An. morulusa* Wang, Chen & Wu, sp. n.

**Figures 11–13.**
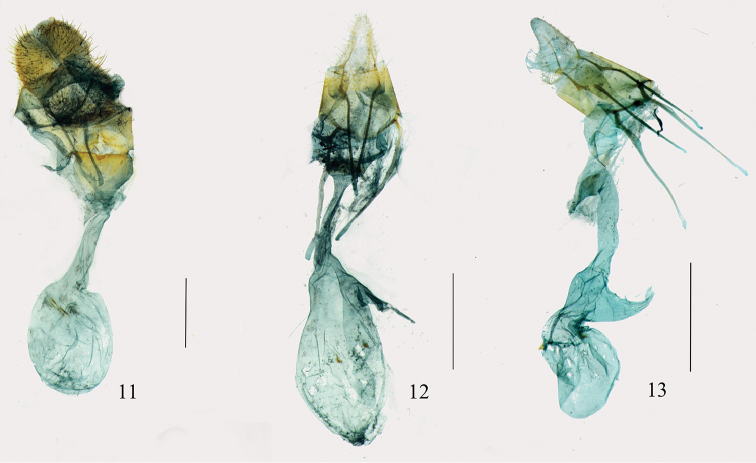
Female genitalia. **11** *Arcanusa sinuosa* (Moore, 1888), comb. n., gen. slide no. Ep538 **12** *Androconia rallusa* Wang, Chen & Wu, sp. n., paratype, gen. slide no. Ep702 **13** *An. morulusa* Wang, Chen & Wu, sp. n., paratype, gen. slide no. Ep692. Scale bars: 1.0 mm.

#### Description.

Adult. Forewing length 9.0–12.0 mm (n=7). Male. Head yellow mixed with fuscous scales; first and second segment of labial palpus with yellow scales; third segment of female slender, and with black scales, approximately 1/3 length of whole labial palpus; third segment of male short; antenna pale yellow scales. Thorax mixed with yellow and small number of fuscous scales. Forewing yellow mixed with fuscous scales; base covered with yellow scales, base 1/3 and 2/3 of costa with a black spot respectively; antemedial line black, indistinct; two black spots located at basal and terminal of cell, respectively. Central area suffused with pale yellow mixed brown; an androconium presents on the discocellular; postmedial line thin, waved, black with yellow edges, outer area covered with yellow scales; cilia mixed with yellow and fuscous. Hindwing paler than forewing and more or less reddish, postmedial line waved. Female. Similar with male, but forewing with less fuscous scales and androconium absent, hindwing redder.

**Male genitalia.** Uncus slender, densely suffused with setae at lateral apex. Gnathos with slender lateral arms, apex hooked. Valva slender, costa lightly sclerotized; sacculus swollen and warped in base. Juxta nearly same width form base to apex, apex bifurcated. Phallus slender.

**Female genitalia.** Ovipositor narrow and pointed at apex, covered with dense setae. Apophysis anterior nearly as same length as apophysis posterior. Antrum membranous. Ductus bursae membranous, slightly shorter than corpus bursae. Ductus seminalis relatively narrow, arising from basal corpus bursae. Corpus bursae nearly elliptic, with two signa, tongue-shaped.

#### Holotype.

♂, **Xizang**: Medog, 1005 m, 15.VIII.2015, Wang Mingqiang, gen. slide. no. Ep537 (IZCAS).

#### Paratypes.

**Fujian**: Wuyishan, Guadun, 1200 m, 1♂, 12.VIII.1979, Song Shimei, gen. slide. no. Ep701 (IZCAS). **Xizang**: Medog, Beibeng, 870 m, 1♀, 17.VIII.2006, Chen Fuqiang, gen. slide. no. Ep702 (IZCAS); Medog, Beibeng, 799 m, 3♀♀, 19.VIII.2015, Wang Mingqiang, gen. slide. no. Ep669, Ep684, Ep689 (IZCAS).

#### Distribution.

China (Fujian, Xizang).

#### Etymology.

The specific name is derived from the Latin “*rallus*” (= thin) in accordance with the thin antemedial line.

### Androconia morulusa

Taxon classificationAnimaliaLepidopteraPyralidae

Wang, Chen & Wu sp. n.

http://zoobank.org/4C53CB46-CF2C-4477-BD1E-72A7EEA9148D

Figs 5–6 , 10 , 13 , 18–19

#### Differential diagnosis.

The species is similar to *An. rallusa* Wang, Chen & Wu, sp. n. in external characters. It can be distinguished from the latter by the darker wings. In male genitalia, the juxta of *An. morulusa* Wang, Chen & Wu, sp. n. is broader than *An. rallusa* Wang, Chen & Wu, sp. n.

**Figures 14–15.**
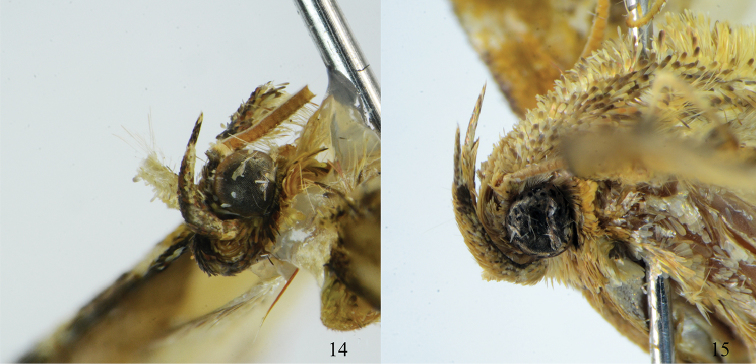
Head of *Arcanusa* Wang, Chen & Wu, gen. n. species. **14** *Ar. apexiarcanusa* Wang, Chen & Wu, sp. n., male, holotype **15** *Ar. sinuosa* (Moore, 1888), comb. n., male.

#### Description.

Adult. Forewing length 10.0–11.5 mm (n=3). Male. Head mixed with black and fuscous scales; first and second of labial palpus segments mixed with yellow and fuscous scales; third segment slender, with black scales; antenna with brown scales. Thorax with fuscous or yellow scales. Forewing covered with blackish-green scales; base area yellow, antemedial line indistinct, central area with a rufous androconium present on the discocellular; postmedial line blurry, waved; outer area black; cilia mixed with yellow and black. Hindwing fuscous and more or less reddish. Female. Similar with male, but forewing with less black scales and androconium absent, hindwing redder.

**Male genitalia.** Uncus slender, densely suffused with setae at lateral apex. Gnathos with slender lateral arms, apex hooked. Valva slender, costa lightly sclerotized; sacculus swollen and warped. Juxta constricted from base to apex, apex bifurcated. Phallus slender, an irregular cornutus present.

**Female genitalia.** Ovipositor narrow, nearly triangle, covered with dense setae. Apophysis anterioris nearly as same length as apophysis posterioris. Antrum and ductus bursae membranous, slightly longer than corpus bursae. Ductus seminalis relatively broad, then extremely constricted, arising basally from corpus bursae. Corpus bursae nearly elliptic, with two signa, nearly triangle.

**Figures 16–19.**
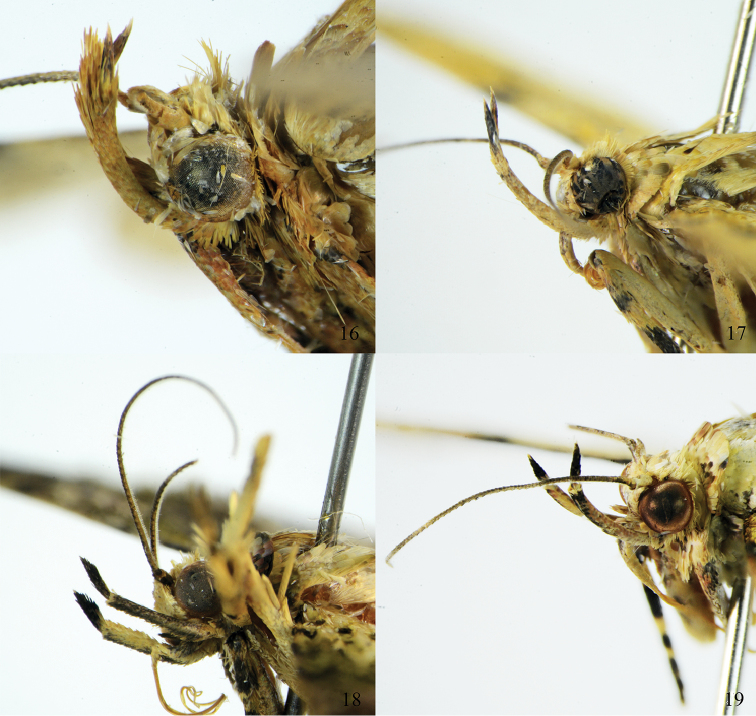
Head of *Androconia* Wang, Chen & Wu, gen. n. species. **16** *An. rallusa* Wang, Chen & Wu, sp. n., male, paratype **17** *An. rallusa* Wang, Chen & Wu, sp. n., female, paratype **18** *An. morulusa* Wang, Chen & Wu, sp. n., male, holotype **19** *An. morulusa* Wang, Chen & Wu, sp. n., female, holotype.

#### Holotype.

♂, **Guangxi**: Maoershan, Gaozhai, 448 m, 13.VIII.2012, Chen Fuqiang, gen. slide. no. Ep691 (IZCAS).

#### Paratypes.

**Guangxi**: Maoershan, Jiuniutang, 1146 m, 1♀, 19.VIII.2012, Chen Fuqiang, gen. slide. no. Ep690 (IZCAS). **Yunnan.** Xiaomengyang, 810 m, 1♀, 31.III.1957, Pu Fuji, gen. slide. no. Ep692 (IZCAS).

#### Distribution.

China (Guangxi, Yunnan).

#### Etymology.

The specific name is derived from the Latin “*morulus*” (= dark-colored) in accordance with the black scales on forewing.

**Figures 20–21.**
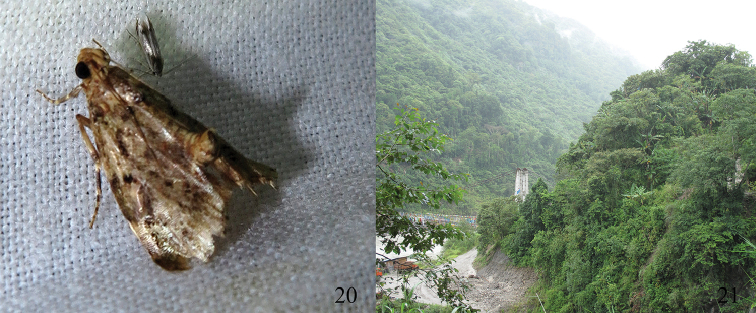
*Androconia rallusa* Wang, Chen & Wu, sp. n., resting position and habitat. **20** Male, holotype **21** Habitat, China, Southeast of Tibet, Medog, 799 m (photo by Mingqiang Wang).

#### Remarks.

The species is sexually dimorphic in color. The male holotype (gen. slide no. Ep691) and a female paratype (gen. slide. no. Ep690) were collected from Maoershan, Guangxi. Although they have different elevations, their localities are adjacent (approximately 3.2 km in straight line). Both of these specimens have the same wing pattern and are thus treated as conspecific.

**Figures 22–23.**
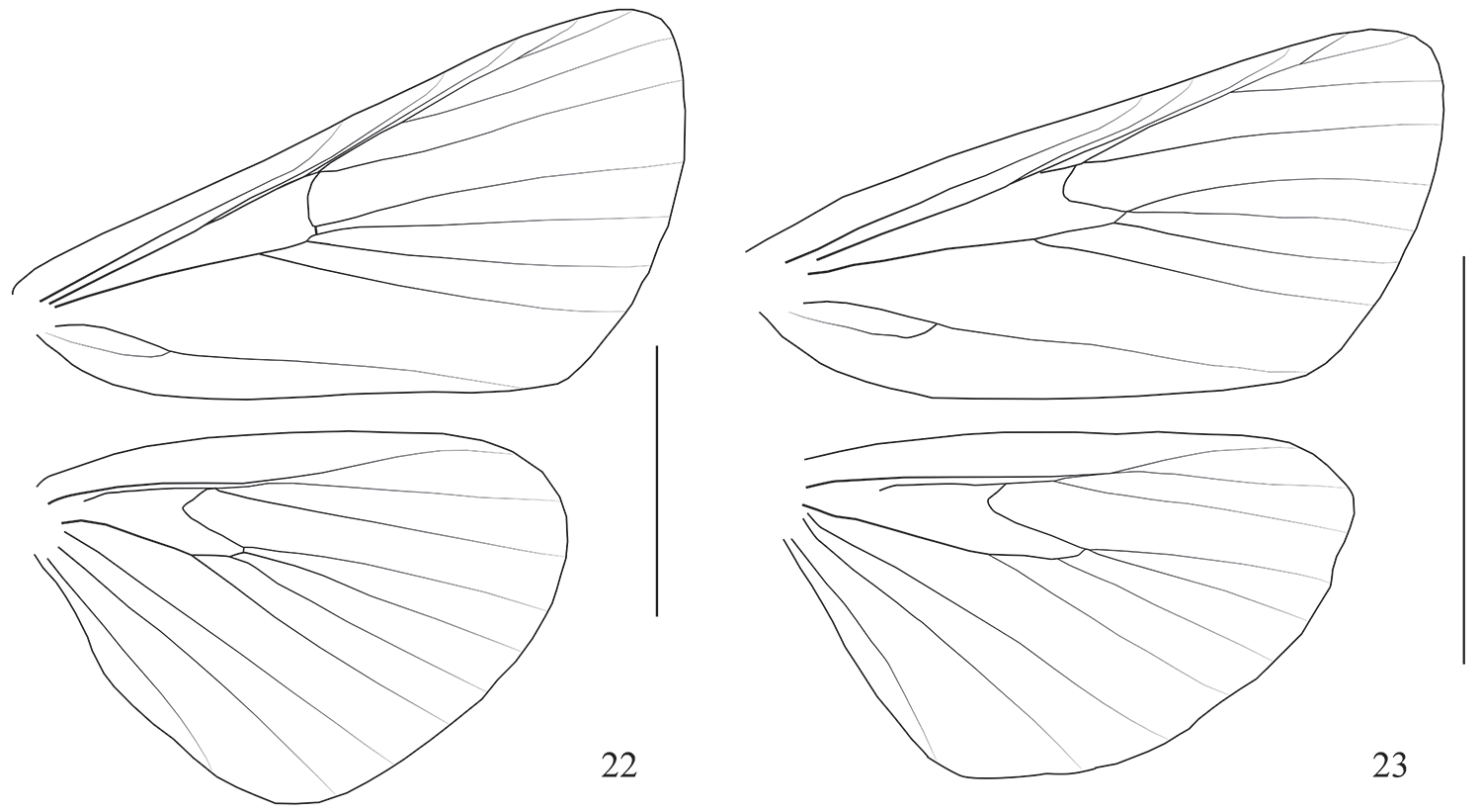
Venation. **22** *Arcanusa sinuosa* (Moore, 1888), comb. n. **23** *Androconia rallusa* Wang, Chen & Wu, sp. n. Scale bars: 5.0 mm.

**Figure 24.**
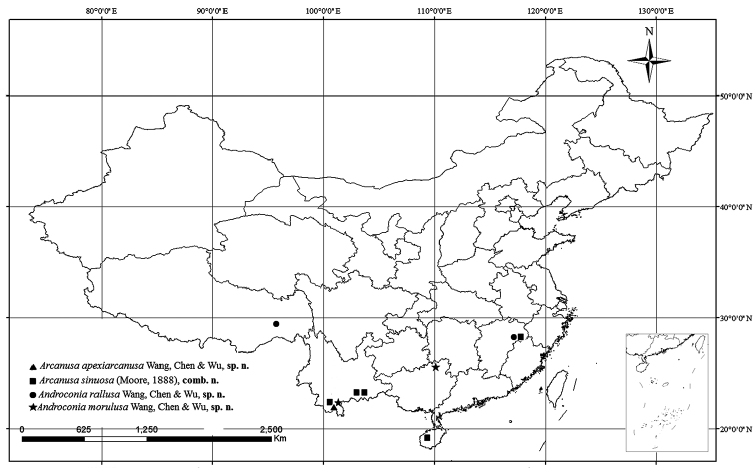
Distribution map of *Arcanusa* Wang, Chen & Wu, gen. n. and *Androconia* Wang, Chen & Wu, gen. n. in China.

## Supplementary Material

XML Treatment for Arcanusa

XML Treatment for Arcanusa apexiarcanusa

XML Treatment for Arcanusa sinuosa

XML Treatment for Androconia

XML Treatment for Androconia rallusa

XML Treatment for Androconia morulusa
